# Preamble and Resolutions in Regard to Cholera, by the New York Academy of Medidine

**Published:** 1866-07

**Authors:** 


### Preamble and Resolutions in regard to Cholera, by the New York Academy of Medidine.

The following resolutions have been sent us through the President, Dr. James Anderson, by order of the Academy, for publication. We are much obliged for opportunity to give publicity to the sentiments of this distinguished medical body:

*Whereas*, The New York Academy of Medicine has endeavored to promote among its own members, and throughout the medical profession, a spirit of exact and practical inquiry into the preventive and remedial treatment of epidemic cholera; therefore, be it

*Resolved*, That this Academy hereby expresses its confidence in the utility of general and specific hygienic measures as the best means of protection against the pestilential prevalence of cholera in any locality where it makes its appearance; and that the most thorough scavenging, cleansing, and disinfection are absolutely necessary means of averting this pestilence in the cities and populous towns of our country at the present time.

*Resolved*, That in the judgment of the Academy the medical profession throughout this country should, for all practical purposes, act and advise in accordance with the hypothesis (or the fact) that the cholera diarrhœa and "rice-water discharges" of cholera patients, are capable, in connexion with well-known localizing conditions, of propagating the cholera poison; and that rigidly enforced precautions should be taken in every case of cholera to permanently disinfect or destroy those ejected fluids by means of active chemical agents; also that with the same object in view, the strictest cleanliness of person and premises should be enforced upon all who have charge of the sick; also, that all privies, water-closets, and cesspools should be kept thoroughly under the control of disinfectants.

*Resolved*, That we regard the nature and causes of cholera infection, so far as the sick or their discharges can propagate it, as being so susceptible of control that there should be no fear or hesitancy in the personal care of the sick and all that pertains to them.

*Resolved*, That immediate and thorough cleansing and disinfection of all persons, clothing, and things that have been exposed to the discharges or persons of the sick with cholera, constitutes the chief end and object of any rational quarantine or external sanitary police regulation against cholera.

*Resolved*, That, for the purposes here mentioned, an external sanitary police is desirable in all great maritime and river towns, but that such sanitary regulations need not seriously embarrass commercial intercourse and the interests of trade.

*Resolved*, That the main source of protection against epidemic cholera at the present time is to be found in the vigilant and effective operation of sanitary measures, municipal, domestic and personal.

*Resolved*, That the New York Academy of Medicine cordially invites the physicians of every city and village throughout our country to urge the immediate adoption of adequate measures of sanitary protection against the introduction and ravages of cholera, and that to this end we pledge our brethren and the public the hearty and continued coöperation of this Academy.

The above resolutions were unanimously adopted by the Academy.

